# Multifraction stereotactic radiotherapy utilizing inhomogeneous dose distribution for brainstem metastases: a single-center retrospective analysis

**DOI:** 10.1093/jrr/rrae057

**Published:** 2024-08-17

**Authors:** Toshiki Ikawa, Naoyuki Kanayama, Hideyuki Arita, Koji Takano, Mio Sakai, Masahiro Morimoto, Kazunori Tanaka, Yutaro Yoshino, Setsuo Tamenaga, Koji Konishi

**Affiliations:** Department of Radiation Oncology, Osaka International Cancer Institute, 3-1-69 Otemae, Chuo-ku, Osaka 541-8567, Japan; Department of Radiation Oncology, Osaka International Cancer Institute, 3-1-69 Otemae, Chuo-ku, Osaka 541-8567, Japan; Department of Neurosurgery, Osaka International Cancer Institute, 3-1-69 Otemae, Chuo-ku, Osaka 541-8567, Japan; Department of Neurosurgery, Osaka International Cancer Institute, 3-1-69 Otemae, Chuo-ku, Osaka 541-8567, Japan; Department of Diagnostic and Interventional Radiology, Osaka International Cancer Institute, 3-1-69 Otemae, Chuo-ku, Osaka 541-8567, Japan; Department of Radiation Oncology, Osaka International Cancer Institute, 3-1-69 Otemae, Chuo-ku, Osaka 541-8567, Japan; Department of Radiation Oncology, Osaka International Cancer Institute, 3-1-69 Otemae, Chuo-ku, Osaka 541-8567, Japan; Department of Radiation Oncology, Osaka International Cancer Institute, 3-1-69 Otemae, Chuo-ku, Osaka 541-8567, Japan; Department of Radiation Oncology, Osaka International Cancer Institute, 3-1-69 Otemae, Chuo-ku, Osaka 541-8567, Japan; Department of Radiation Oncology, Osaka International Cancer Institute, 3-1-69 Otemae, Chuo-ku, Osaka 541-8567, Japan

**Keywords:** brain neoplasms, brain stem, radiosurgery, toxicity, linear accelerator, fractionated

## Abstract

Brainstem metastases are challenging to manage owing to the critical neurological structures involved. Although stereotactic radiotherapy (SRT) offers targeted high doses while minimizing damage to adjacent normal tissues, the optimal dose fractionation remains undefined. This study evaluated the efficacy and safety of multifraction SRT with an inhomogeneous dose distribution. This retrospective study included 31 patients who underwent 33 treatments for 35 brainstem lesions using linear accelerator-based multifraction SRT (30 Gy in five fractions, 35 Gy in five fractions or 42 Gy in 10 fractions) with an inhomogeneous dose distribution (median isodose, 51.9%). The outcomes of interest were local failure, toxicity and symptomatic failure. The median follow-up time after brainstem SRT for a lesion was 18.6 months (interquartile range, 10.0–24.3 months; range, 1.8–39.0 months). Grade 2 toxicities were observed in two lesions, and local failure occurred in three lesions. No grade 3 or higher toxicities were observed. The 1-year local and symptomatic failure rates were 8.8 and 16.7%, respectively. Toxicity was observed in two of seven treatments with a gross tumor volume (GTV) greater than 1 cc, whereas no toxicity was observed in treatments with a GTV less than 1 cc. No clear association was observed between the biologically effective dose of the maximum brainstem dose and the occurrence of toxicity. Our findings indicate that multifraction SRT with an inhomogeneous dose distribution offers a favorable balance between local control and toxicity in brainstem metastases. Larger multicenter studies are needed to validate these results and determine the optimal dose fractionation.

## INTRODUCTION

Brainstem metastases are relatively uncommon [1], and their management is challenging. Surgery for brainstem metastases poses challenges owing to the presence of vital neurological tissues in the brainstem [2]; thus, radiotherapy plays an important role in the management of brainstem metastases. Stereotactic radiotherapy (SRT) is a proven method for treating brain metastases [3, 4]. It delivers high doses precisely to the target volume and minimizes the dose to the surrounding normal tissue. Based on the results of RTOG 9005, the recommended maximum tolerated doses for single-fraction SRT are 24, 18 and 15 Gy for tumors with maximum diameters of <20, 21–30 and 31–40 mm, respectively [5]. The study involved patients with recurrent primary brain tumors or recurrent metastases that had previously been irradiated. However, the optimal prescription method and dose fractionation of SRT for brainstem metastases remain unknown because these patients have often been excluded from clinical trials, and most available data on SRT for brainstem metastases are limited to retrospective studies with a limited number of patients [2, 6].

When considering the optimal dose fractionation for brainstem metastases, we face the dilemma of balancing tumor control against toxicity. The brainstem is composed of neurologically critical tissues, and failure to control brainstem lesions can lead to severe neurological decline [2]. Prescribing a high radiation dose may improve tumor control but may cause brainstem damage and severe neurological toxicities [2]. The following two methods can reduce toxicity while maintaining tumor control in brainstem metastases. First, multifraction SRT reportedly enhances local control and lowers the risk of brain necrosis in large tumors compared with single-fraction SRT [7, 8]. Second, inhomogeneous dose distribution increases the dose to the target volume while sharply reducing it outside the target in the brain parenchyma [9–11]. Since 2020, our institution has been using multifraction SRT with an inhomogeneous dose distribution for brain metastases, including brainstem metastases. In this study, we evaluated tumor control, toxicity and symptomatic failure (as a comprehensive outcome of tumor control and toxicity) associated with this treatment approach.

## MATERIALS AND METHODS

### Study design and patients

This retrospective study was approved by the ethics committee of Osaka International Cancer Institute (approval number 21150) and conducted according to the tenets of the Declaration of Helsinki. All patients provided written informed consent for the use of their data for clinical research before radiotherapy administration and had the opportunity to opt out of the study. Multifraction SRT with an inhomogeneous dose distribution for brainstem metastases was initiated in September 2020 at our institution. From our electronic database, 32 consecutive patients who underwent SRT for brainstem metastases between September 2020 and December 2022 were identified.

### Stereotactic radiotherapy protocol and patient follow-up

SRT was performed as described previously [9, 12, 13]. All patients were immobilized using a thermoplastic mask, and planning computed tomography was performed using an iodine contrast agent unless medically contraindicated. Gross tumor volume (GTV) was delineated using T1-weighted gadolinium-enhanced magnetic resonance imaging (MRI). The planning target volume (PTV) was generated by adding an isotropic margin of 1 mm to the GTV. A decrease in the margin to 0 mm was permitted based on the patient’s condition.

We ordinarily prescribed 30 or 35 Gy in five fractions for brainstem metastases but allowed 42 Gy in 10 fractions at the radiation oncologist’s discretion. The dose was prescribed to cover 95 or 99% (D95 or D99) of the combined PTVs in the brainstem and non-brainstem areas. The goals of planning optimization are listed in Table 1, but the procedure may still be deemed acceptable even if they were not achieved. The brainstem dose was evaluated using the following parameters:

**Table 1:** Dose objectives for SRT planning

Dose/fractions	PTV D95 or D99	GTV D98–99	GTV Dmax	V12Gy-eq of Brain−GTV
30 Gy/5 or 35 Gy/5	30 Gy or 35 Gy	43 Gy	≥50 Gy	Minimize V25Gy
42 Gy/10	42 Gy	52.5 Gy	≥60 Gy	Minimize V33Gy

Abbreviations: PTV, planning target volume; GTV, gross tumor volume; Dmax, maximum dose; Vx, volume receiving greater than x Gy; Dx, dose to the highest x% of volume.

- V12Gy-equivalent of Brain minus GTV (V12Gy-eq of Brain−GTV): brain volume within 10 mm in all directions from the brainstem GTV, excluding the brainstem GTV, receiving ≥12 Gy in a single fraction. This corresponds to Brain−GTV receiving ≥25 Gy in five fractions and ≥ 33 Gy in 10 fractions, using a linear-quadratic model [14] with an α/β ratio of 2.
- D0.03cc of Brain−GTV: dose to the highest 0.03 cc of Brain−GTV. This represents the maximum dose delivered to the brainstem.

All treatments were performed using automated non-coplanar volumetric-modulated arc therapy (HyperArc; Varian Medical Systems, Palo Alto, CA, USA) or non-coplanar dynamic conformal arc therapy with a linear accelerator equipped with a 2.5-mm multileaf collimator (TrueBeam STx or Edge; Varian Medical Systems). Corticosteroids were administered when neurological symptoms were present or when peritumoral brain edema was significant, and there was a risk of symptom emergence. Follow-up examinations, including clinical examinations and MRI, were conducted at least every 4 months during the first 2 years after SRT initiation and at least every 6 months thereafter.

### Outcome evaluation

First, toxicity was observed at the level of the lesion. Toxicity data were collected from medical records. Alopecia and mild symptoms such as mild headache and nausea were not considered as toxicity. Toxicity grades were determined based on the CTCAE version 5.0. Second, local failure (LF) of brainstem lesions was observed at the level of the lesion. LF was defined as tumor progression according to the Response Assessment in Neuro-Oncology Brain Metastases (RANO-BM) guidelines [15]. This definition was applied to each brainstem lesion. The detailed differential diagnosis between tumor progression and brain necrosis based on imaging findings has been previously reported [9]. However, distinguishing between these conditions is challenging. Therefore, two radiation oncologists and a neurosurgeon conducted a comprehensive assessment of the post-treatment course and reached a consensus on distinguishing between tumor progression and brain necrosis. Third, symptomatic failure was evaluated. The original definition of symptomatic failure, as proposed by Nakamura [16], is the occurrence of new neurological symptoms or worsening of existing neurological symptoms owing to the progression of brainstem lesions or toxicity. We also included cases that required treatment because of the progression of brainstem lesions or brainstem-associated toxicity. These interventions included the initiation or change of chemotherapy, administration of steroids or bevacizumab, surgery, radiotherapy and transition to palliative care following the discontinuation of treatment. Symptomatic failure was observed at the patient level.

### Statistical analyses

Overall survival (OS) was measured at the patient level from the first brainstem SRT to the last follow-up or death using the Kaplan–Meier method. The cumulative incidence of LF was measured from brainstem SRT initiation to the radiological observation of tumor progression in a treated lesion. The cumulative incidence of symptomatic failure was measured from brainstem SRT initiation to the date of the occurrence of new neurological symptoms, worsening of existing neurological symptoms, or therapeutic interventions. Cumulative incidence was estimated using the cumulative incidence function, accounting for death as a competing risk [17]. All analyses were performed using the R software (version 4.2.3) (R Foundation for Statistical Computing, Vienna, Austria).

## RESULTS

### Patient, tumor and treatment characteristics

Among the 32 patients who underwent SRT for brainstem metastases, one was excluded from the analysis because, at the radiation oncologist’s discretion, the dose was prescribed for a brainstem lesion that exhibited an isodose level (PTV D95/max dose × 100) above 80% (i.e. lesions demonstrating a homogeneous dose distribution). The isodose distribution for all lesions is presented in Supplementary Fig. 1. Among the remaining 31 patients, one had two brainstem lesions treated simultaneously with a single session of SRT, one had three brainstem lesions treated with two separate sessions of SRT and one underwent SRT for a brainstem lesion and later received SRT again for the LF of the lesion. In total, 31 patients were included in the study, with 33 SRT treatments and 35 brainstem lesions.

The patient and treatment characteristics of the 33 SRT are shown in Table 2. Prescribed doses included 30 Gy in five fractions for 15 (45.5%) treatments, 35 Gy in five fractions for 14 (42.4%) treatments and 42 Gy in 10 fractions for four (12.1%) treatments. The most common primary cancer type was non-small cell lung cancer, accounting for 15 (45.5%) treatments, followed by small cell lung cancer in five (15.2%) and breast cancer in four (12.1%). The median number of brain metastases treated simultaneously per treatment was 4 (range: 1–52). Patients with three (9.1%) treatments had previously undergone whole-brain radiotherapy, and patients with seven (21.2%) had brainstem-related neurological symptoms before treatment.

**Table 2:** Patient and treatment characteristics

Characteristic	*n* = 33
Age, years	67 (57, 73) [36–86]
Sex	
Female	16 (48.5%)
Male	17 (51.5%)
Performance status	
0–1	28 (84.8%)
2–3	5 (15.2%)
Prescribed dose/number of fractions	
30 Gy in 5 fractions	15 (45.5%)
35 Gy in 5 fractions	14 (42.4%)
42 Gy in 10 fractions	4 (12.1%)
PTV margin, mm	
0	2 (6.1%)
1	31 (93.9%)
Primary cancer	
Lung, non-small cell	15 (45.5%)
Lung, small cell	5 (15.2%)
Breast	4 (12.1%)
Ovary	3 (9.1%)
Kidney	2 (6.1%)
Others	4 (12.1%)
Median number of metastases	4 (1, 10) [1–52]
Number of metastases	
1	9 (27.3%)
2–4	11 (33.3%)
5–9	3 (9.1%)
10–19	6 (18.2%)
20–52	4 (12.1%)
Prior history of whole-brain radiotherapy	3 (9.1%)
Prior history of SRT	14 (42.4%)
Re-irradiation of brainstem lesions previously treated with SRT	1 (3.0%)
Presence of brainstem-associated neurological signs	7 (21.2%)
Use of corticosteroids	11 (33.3%)
Receipt of cytotoxic agents^a^	13 (39.4%)
Receipt of molecularly targeted agents with anti-VEGF activity^a^	4 (12.1%)
Receipt of other molecularly targeted agents^a^	8 (24.2%)
Receipt of immune checkpoint inhibitors^a^	3 (9.1%)

Data are presented as the median (interquartile range) (minimum–maximum) or as *n* (%). Abbreviations: PTV, planning target volume; SRT, stereotactic radiotherapy; VEGF, vascular endothelial growth factor.

^a^Receipt of treatment during or within 1 month before SRT.

The characteristics of the 35 brainstem lesions and their dosimetric parameters are listed in Table 3. The tumor locations included 11 (31.4%) lesions in the midbrain, 23 in the pons (65.7%) and one in the medulla (2.9%). The median GTV volume was 0.20 cc (interquartile range [IQR], 0.03–0.68 cc; range, 0.004–7.69 cc). The median isodose was 51.9% (IQR, 47.0–60.2%; range, 42.0–70.6%). The detailed dosimetric parameters for each prescribed dose are presented in Supplementary Tables 1–3. The median follow-up time after initial brainstem SRT at the patient level was 17.4 months (IQR, 9.2–24.0 months). The 1-year overall survival rate after initial brainstem SRT was 67.7% (95% confidence interval [CI], 53.1–86.4%; Kaplan–Meier curve shown in Supplementary Fig. 2).

**Table 3:** Characteristics of the brainstem lesions and dosimetric parameters

Characteristic	*n* = 35
Tumor location	
Midbrain	11 (31.4%)
Pons	23 (65.7%)
Medulla	1 (2.9%)
Median GTV volume, cc	0.20 (0.030, 0.68) [0.004–7.69]
GTV volume, cc	
<0.1	13 (37.1%)
0.1–1	15 (42.9%)
1–4	3 (8.6%)
4–7.7	4 (11.4%)
Isodose (PTV D95/Max dose × 100), %	51.9 (47.0, 60.2) [42.0–70.6]
V12Gy-eq of Brain−GTV, cc	0.64 (0.28, 1.27) [0.05–4.90]

Data are presented as the median (interquartile range) (minimum–maximum) or as *n* (%). Abbreviations: GTV, gross tumor volume; PTV, planning target volume.

### Toxicities

**Fig. 1:**
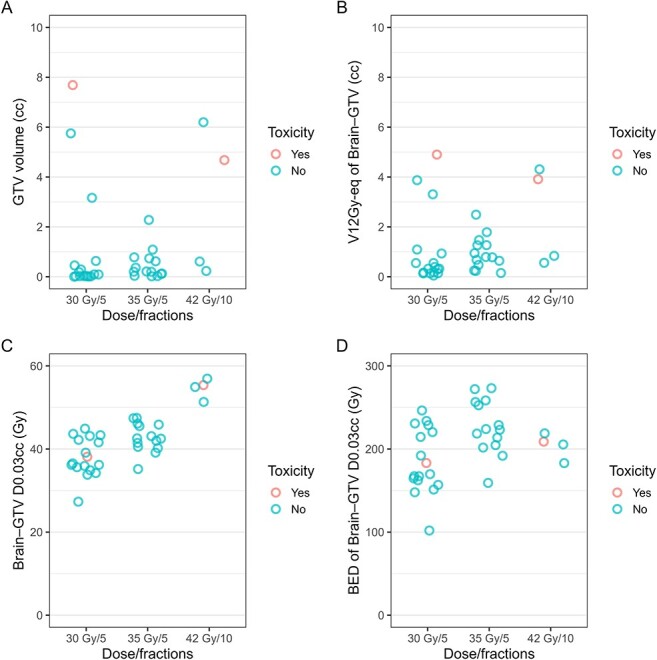
Relationships among specific dosimetric parameters, dose fractionation and toxicity, illustrating **A**: GTV volume, **B**: V12Gy-eq of Brain−GTV, **C**: D0.03cc of Brain−GTV and **D**: biologically effective dose (BED, α/β ratio = 2) of Brain−GTV D0.03cc.

The median follow-up time after brainstem SRT at the lesion level was 18.6 months (IQR, 10.0–24.3 months; range, 1.8–39.0 months). No toxicity was observed during or immediately after the SRT. Toxicities were observed in two of the 35 lesions (5.7%); details on these lesions and SRT treatments are provided in Table 4. In one case, SRT of 42 Gy in 10 fractions was administered for a 4.7-cc lesion in the midbrain. The patient had difficulty walking owing to left-sided muscle weakness, but after SRT, the symptoms improved. At 9.8 months post-treatment, the patient experienced a recurrence of left-sided muscle weakness but remained ambulatory. MRI findings suggested brain necrosis (Supplementary Fig. 3), leading to a diagnosis of Grade 2 toxicity. The patient continued chemotherapy, including bevacizumab, and started steroids, which mildly improved the symptoms. These treatments continued until the patient's death owing to worsening systemic metastases. In another case, SRT of 30 Gy in five fractions was administered for a 7.7-cc lesion in the pons. The patient had left lower limb weakness and diplopia, but after SRT, the symptoms improved. At 11 months post-treatment, the patient experienced a recurrence of diplopia (one-and-a-half syndrome). MRI findings suggested brain necrosis, leading to grade 2 toxicity. The patient continued chemotherapy, including bevacizumab, but the symptoms persisted. Figure 1A–D shows the relationship among specific dosimetric parameters, dose fractionation and toxicity. Specifically, A: GTV volume, B: V12Gy-eq of Brain−GTV, C: D0.03cc of Brain−GTV and D: biologically effective dose (BED, α/β ratio = 2) of Brain−GTV D0.03cc. Toxicity was observed in two out of seven (28.6%) treatments with GTV volumes greater than 1 cc, whereas no toxicity was observed in treatments with GTV volumes less than 1 cc. No toxicity was observed in treatments with V12Gy-eq of Brain−GTV less than 2 cc. No clear association was found between the BED of Brain−GTV D0.03cc and the occurrence of toxicity.

**Table 4:** Details of toxicity, lesion characteristics and dosimetric parameters in patients who experienced toxicity

Primary cancer	Tumor location	Dose/fractions	GTV volume, cc	V12Gy-eq of Brain−GTV, cc	D0.03cc of Brain−GTV, Gy	History of whole-brain radiotherapy	Receipt of systemic therapy	Time to toxicity, months	Toxicity
Cervix	Midbrain	42 Gy/10	4.7	3.9	55.4	No	No	9.8	Central nervous system necrosis grade 2; muscle weakness left-sided (hemiparesis) grade 2
Lung, non-small	Pons	30 Gy/5	7.7	4.9	38.1	No	No	11.0	Central nervous system necrosis grade 2; nervous system disorders—others (one-and-a-half syndrome) grade 2

Abbreviations: GTV, gross tumor volume. Systemic therapy includes cytotoxic chemotherapeutic agents, molecularly targeted agents and immune checkpoint inhibitors administered during or within 1 month before stereotactic radiotherapy.

### Local failure

**Fig. 2:**
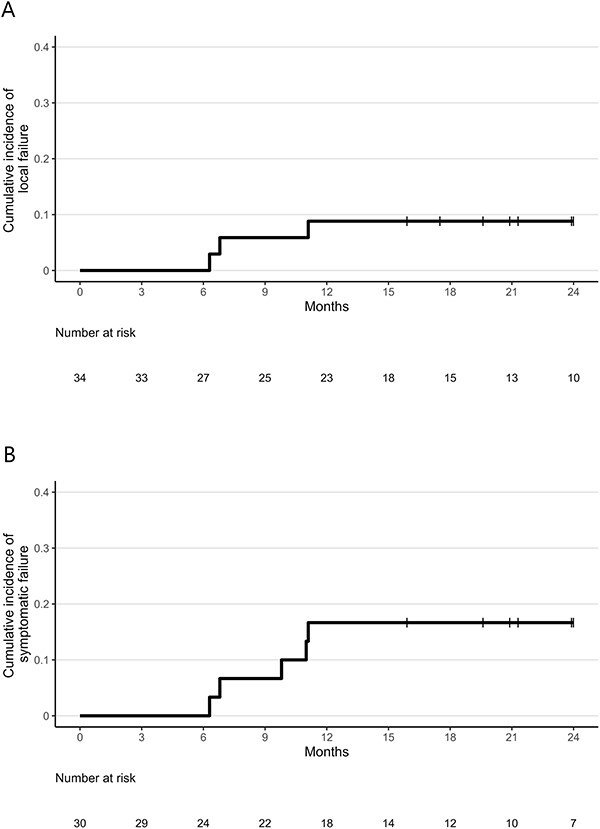
Cumulative incidence of local failure (**A**) and symptomatic failure (**B**).

One patient who did not undergo MRI after SRT was excluded. LF occurred in three lesions (8.8%), with details of these lesions described in Table 5. Illustrative MRI findings for a case of LF are presented in Supplementary Fig. 4. The 1-year LF rate was 8.8% (95% CI, 2.2–21.4%; Fig. 2A). All three cases were treated with 30 Gy in 5 fractions. Patients 1 and 2 discontinued the treatment and transitioned to palliative care. Patient 3 received re-irradiation with SRT of 42 Gy in 10 fractions, achieving local control for 27.2 months without toxicities.

**Table 5:** Characteristics of lesions and dosimetric parameters in patients who experienced local failure

No.	Primary cancer	Number of metastases	Tumor location	Time to local failure, months	Dose/ fractions	PTV margin, mm	GTV volume, cc	PTV D95, Gy	GTV D99, Gy	Isodose, %
1	Lung, small cell	18	Midbrain	6.3	30 Gy/5	0	0.028	32.2	30.8	70.6
2	Lung, small cell	1	Pons	6.8	30 Gy/5	1	3.2	32.1	40.4	45.0
3	Breast	8	Pons	11.1	30 Gy/5	1	0.018	34.6	46.2	62.1

Abbreviations: PTV, planning target volume; GTV, gross tumor volume

### Symptomatic failure

Three patients developed symptomatic failure owing to LF and two owing to toxicity. The 1-year symptomatic failure rate was 16.7% (95% CI, 5.9–32.2%; Fig. 2B).

## DISCUSSION

The management of brainstem metastases is a major therapeutic challenge, balancing the need for local control and toxicity. This study explored the use of multifraction SRT with an inhomogeneous dose distribution to optimize treatment efficacy while minimizing adverse effects. However, the optimal dose fractionation for brainstem metastases has not yet been established. Regarding single-fraction SRT, a large multicenter retrospective study of 547 patients with brainstem metastases reported a 1-year survival rate of 32.7%, a 1-year local control rate of 82% and a grade ≥ 3 toxicity rate of 7.4% [2]. The study indicated that lesions smaller than 0.1 cc did not result in severe adverse events. In a meta-analysis of SRT for brainstem metastases, in which the majority of patients underwent single-fraction SRT, a 1-year survival rate of 33%, a 1-year local control rate of 86% and a grade ≥ 3 toxicity rate of 2.4% were reported [6]. The study concluded that for lesions smaller than 1 cc, treatment toxicity remained within acceptable limits. Thus, in determining dose fractionation, tumor volume is an important factor in the incidence of severe adverse events.

Only a few reports exist on multifraction SRT for brainstem metastases, primarily retrospective analyses with limited case numbers [16,18–23]. Nicosia *et al.* [24] conducted a relatively large multicenter retrospective study that analyzed 111 brainstem metastases in 105 patients treated with single-fraction or multifraction SRT. Multifraction SRT was primarily administered to lesions >10 mm at doses of 14–32 Gy in two–five fractions, with 53 of the 111 brainstem metastases receiving multifraction SRT. They reported a 1-year local progression-free rate of 90.4% with no grade ≥ 3 toxicity.

We investigated the efficacy and safety of SRT at doses of 30 Gy in five fractions, 35 Gy in five fractions, or 42 Gy in 10 fractions at a median isodose level of 51.9% for treating brainstem metastases. We employed multifraction SRT even for small-volume lesions to minimize SRT toxicity in long-term survivors. Our study showed a favorable 1-year overall survival rate of 67.7% after initial SRT for brainstem metastases compared to previous reports [2, 6]. This improved survival for patients with brainstem lesions may be attributed to recent advancements in oncology, such as molecular targeted therapies and immunotherapy [25]. Furthermore, the widespread use of MRI screening may have increased the detection rate of asymptomatic brain metastases, leading to better survival outcomes [25]. The 1-year LF rate was 8.8%, and the 1-year symptomatic failure rate was 16.7%, with no grade ≥ 3 toxicities observed. The majority of patients in this study exhibited a maximum dose (D0.03cc) to the brainstem exceeding 31 Gy in five fractions or 38 Gy in 10 fractions, which were the proposed constraints for the brainstem dose [26–28]. Notably, the toxicity levels were within acceptable limits. Our results suggest that fractionated and inhomogeneous prescriptions may offer high local control and acceptable toxicity in SRT for brainstem metastases despite exceeding traditional brainstem dose constraints. Lehrer *et al.* reported similar findings, suggesting that the maximum brainstem dose may not be an ideal parameter for considering toxicity in Gamma Knife treatment of brain metastases or arteriovenous malformations within or abutting the brainstem [29]. They concluded that an increase in mean brainstem dose, D05 and D95% to the brainstem is associated with post-treatment complications.

As previously mentioned, tumor volume is an important factor associated with the occurrence of toxicity. We observed no toxicity in patients with tumor volumes less than 1 cc, suggesting that treatment toxicity in brainstem SRT is acceptable for tumors smaller than 1 cc. Toxicity was observed in patients with larger lesions, specifically those with tumor volumes of 4.7 cc and 7.7 cc. Furthermore, we used V12Gy-eq of Brain−GTV as a parameter for the brainstem dose. No toxicity was observed in the volumes less than 2 cc. In the non-brainstem area, an increase in V12Gy-eq of Brain−GTV is associated with brain necrosis [30]. Dose fractionation and GTV-PTV margins vary among studies. This variation makes it difficult to compare post-treatment toxicity across studies based solely on prescription dose and tumor volume. Including V12Gy-eq of Brain−GTV can improve comparability across studies. However, owing to the limited number of large lesions, further investigation is needed to determine the tumor volume and V12Gy-eq cutoff values at which SRT for brainstem lesions exhibits acceptable toxicity. Other factors associated with severe toxicity include margin dose, prior or concurrent whole-brain radiotherapy and brainstem metastasis of melanoma [2, 6]. In this study, only three patients had a history of whole-brain radiotherapy, and there were no cases of melanoma brainstem metastasis. Additionally, only one patient had medullary metastasis. Therefore, our results cannot be generalized to these patients, and caution is needed when interpreting the study's findings.

LF was observed in three lesions treated with 30 Gy in five fractions. To improve local control, a dose of 35 Gy in five fractions or 42 Gy in 10 fractions might be more appropriate. A large-scale multicenter retrospective study reported that increased margin doses are associated with local control of brainstem metastases [2]. Conversely, a meta-analysis of SRT for brainstem metastases did not find a significant association between radiation dose and local control [6]. These inconsistent results may be affected by the difficulty of differentiating tumor recurrence from adverse radiation effects, including radiation necrosis, based on imaging studies [31, 32]. Decisions in our study were made after discussions among two radiation oncologists and one neurosurgeon (illustrative cases for suspected brain necrosis and LF are shown in Supplementary Figs 3 and 4); however, there remains a potential risk of misclassification. Therefore, symptomatic failure [16] was used as a comprehensive indicator of both local control and toxicity. Considering the presence of vital neurological tissues in the brainstem, a balanced treatment approach that considers both local control and toxicity is crucial. Given the possibility that the differentiation criteria may vary among reporters, it is advisable to report indicators such as symptomatic failure, in addition to local control and toxicity, when documenting treatment outcomes for brainstem metastases.

In conclusion, our findings indicate that multifraction SRT with an inhomogeneous dose distribution is a promising approach for managing brainstem metastases, achieving a favorable balance between local control and toxicity risk. However, our study has limitations, including its single-center, retrospective observational design and small sample size. Larger multicenter studies that employ a comprehensive assessment of local control and toxicity are needed to validate these results and determine the optimal dose fractionation.

## Supplementary Material

Supplemental_data_revised_rrae057
