# Supplementary material for: Multifraction stereotactic radiotherapy utilizing inhomogeneous dose distribution for brainstem metastases: a single-center retrospective analysis
Source: J Radiat Res. 2024 Aug 17;65(5):658–66. doi: 10.1093/jrr/rrae057 (PMC11420839; doi:10.1093/jrr/rrae057)
Supplement: Supplemental_data_revised_rrae057 [file supplemental_data_revised_rrae057.docx]

**Supplementary Table 1.** Characteristics of brainstem lesions and dosimetric parameters of stereotactic radiotherapy (SRT) of 30 Gy in five fractions.

| Characteristic | N = 17 |
| --- | --- |
| Tumor location |  |
| Midbrain | 5 (29.4%) |
| Pons | 12 (70.6%) |
| Medulla | 0 (0.0%) |
| Median GTV volume, cc | 0.09 (0.018, 0.45) [0.004–7.69] |
| PTV D95, Gy | 31.6 (30.4, 32.1) [29.8–34.6] |
| GTV D99, Gy | 38.8 (36.0, 41.1) [30.8–46.2] |
| Max dose, Gy | 59.5 (48.1, 68.0) [44.0–76.0] |
| Isodose (PTV D95/Max dose × 100), % | 52.9 (45.0, 67.8) [42.0–70.6] |
| V25Gy of Brain−GTV, cc | 0.32 (0.17, 0.93) [0.05–4.90] |
| D0.03cc of Brain−GTV, Gy | 36.5 (35.6, 42.2) [27.3–44.9] |
| Data are presented as n (%) or median (interquartile range) [minimum–maximum].  Abbreviations: GTV, gross tumor volume; PTV, planning target volume | |

**Supplementary Table 2.** Characteristics of brainstem lesions and dosimetric parameters of SRT of 35 Gy in five fractions.

| Characteristic | N = 14 |
| --- | --- |
| Tumor location |  |
| Midbrain | 5 (35.7%) |
| Pons | 8 (57.1%) |
| Medulla | 1 (7.1%) |
| Median GTV volume, cc | 0.21 (0.12, 0.71) [0.017–2.28] |
| PTV D95, Gy | 35.0 (34.3, 35.0) [33.5–36.2] |
| GTV D99, Gy | 44.0 (41.6, 45.3) [35.1–47.4] |
| Max dose, Gy | 66.3 (61.5, 69.1) [53.1–76.4] |
| Isodose (PTV D95/Max dose × 100), % | 52.2 (50.1, 55.3) [45.8–68.2] |
| V25Gy of Brain−GTV, cc | 0.79 (0.51, 1.27) [0.15–2.49] |
| D0.03cc of Brain−GTV, Gy | 42.6 (40.8, 45.8) [35.2–47.5] |
| Data are presented as n (%) or median (interquartile range) [minimum–maximum].  Abbreviations: GTV, gross tumor volume; PTV, planning target volume | |

**Supplementary Table 3.** Characteristics of brainstem lesions and dosimetric parameters of SRT of 42 Gy in 10 fractions.

| Characteristic | N = 4 |
| --- | --- |
| Tumor location |  |
| Midbrain | 1 (25.0%) |
| Pons | 3 (75.0%) |
| Medulla | 0 (0.0%) |
| Median GTV volume, cc | 2.64 [0.23–6.20] |
| PTV D95, Gy | 42.0 [42.0–43.3] |
| GTV D99, Gy | 53.3 [49.3–55.7] |
| Max dose, Gy | 83.7 [80.2–90.6] |
| Isodose (PTV D95/Max dose × 100), % | 50.2 [46.3–54.0] |
| V33Gy of Brain−GTV, cc | 2.38 [0.56–4.31] |
| D0.03cc of Brain−GTV, Gy | 55.2 [51.3–56.9] |
| Data are presented as n (%) or median [minimum–maximum].  Abbreviations: GTV, gross tumor volume; PTV, planning target volume | |


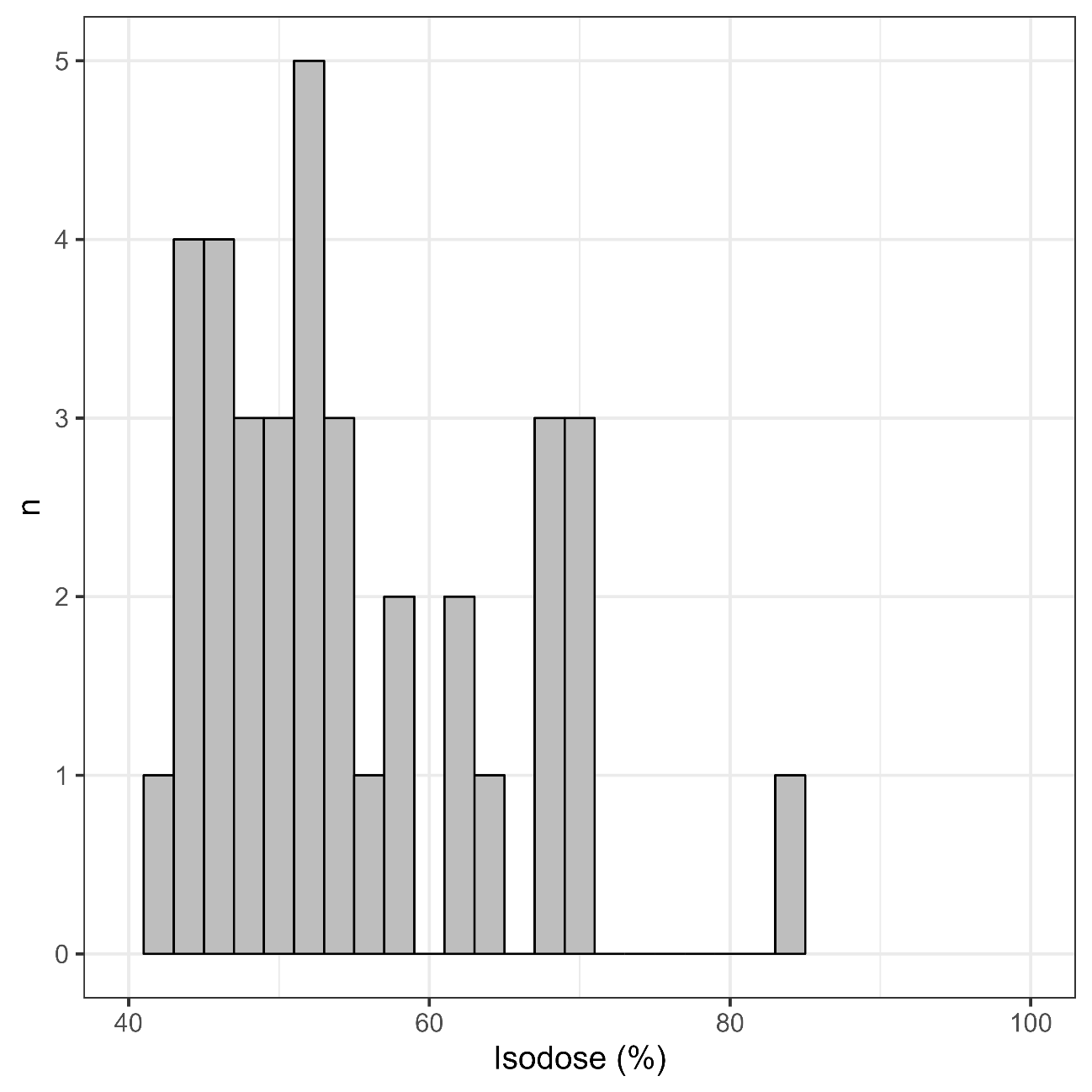
 **Supplementary Fig. 1.** Histogram of the isodose distribution (PTV D95/max dose × 100)


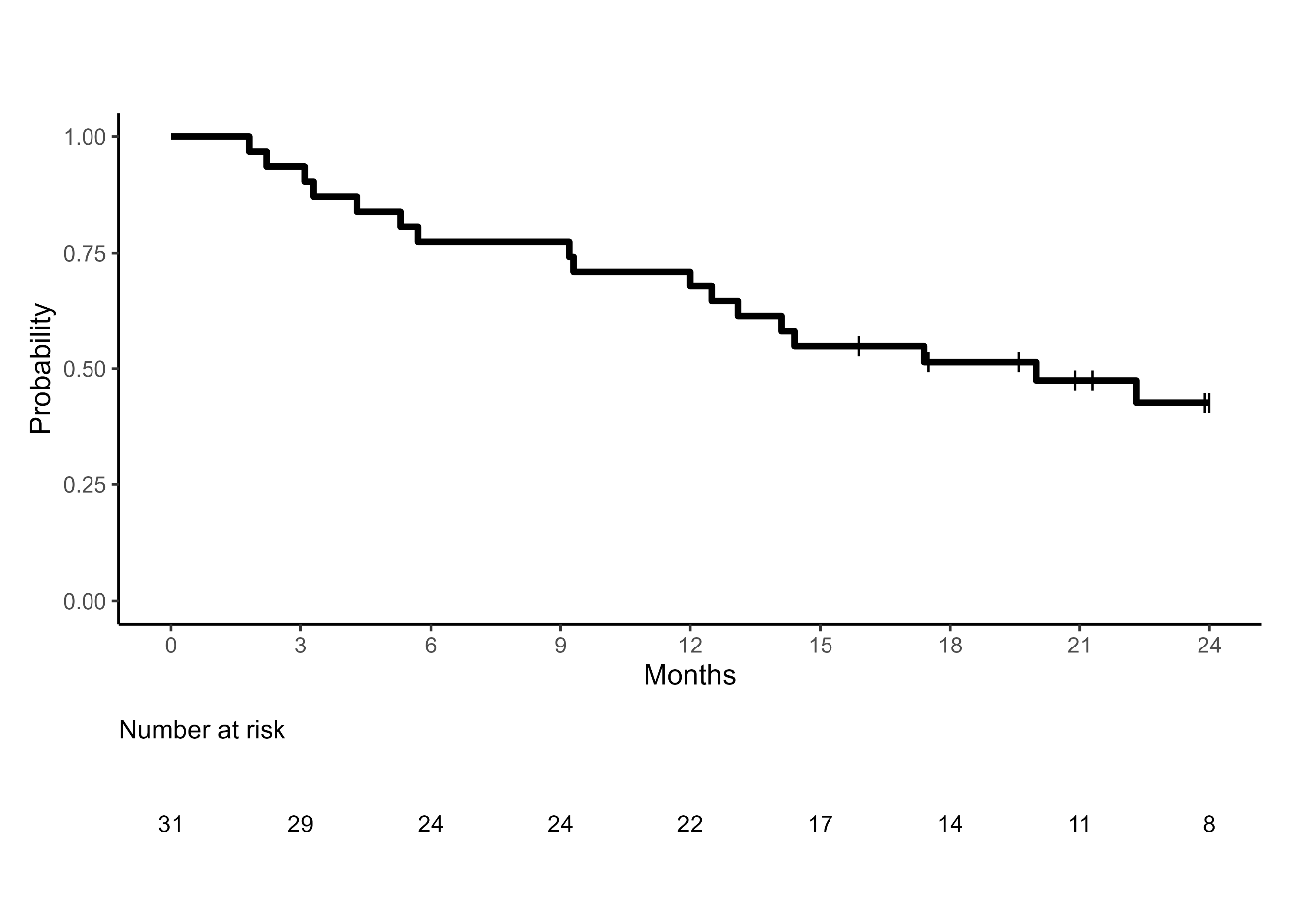


**Supplementary Fig. 2.** Overall survival rate after initial SRT for brainstem metastases


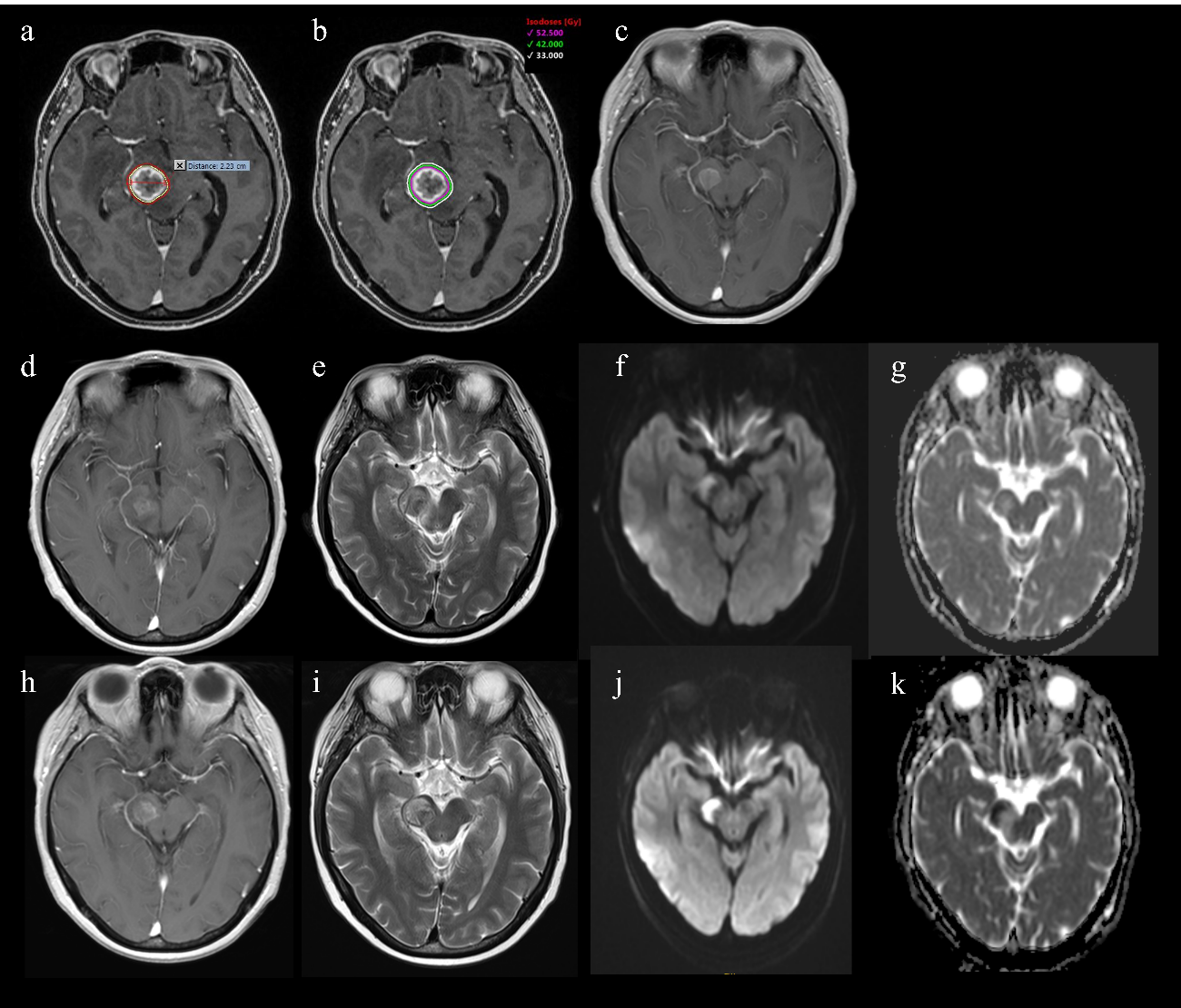


**Supplementary Fig. 3**. A case of midbrain metastasis from cervical cancer treated with stereotactic radiotherapy (SRT) of 42 Gy in 10 fractions, presenting with suspected brain necrosis.

(a) Pre-SRT axial contrast-enhanced (CE)-T1-weighted image showing the GTV (yellow line) and PTV (red line).

(b) Dose distribution of SRT, depicting 52.5 Gy (purple line), 42 Gy (green line), and 33 Gy (white line).

(c) CE-T1-weighted image acquired 4 months post-treatment, indicating a decrease in lesion size compared with pre-SRT image.

(d), (e), (f), (g) CE-T1, T2, and diffusion-weighted images, and apparent diffusion coefficient (ADC) map acquired 9 months post-treatment.

(h), (i), (j), (k) acquired 11 months post-treatment (after steroid administration).

The T2-weighted image at 9 months post-treatment shows an increase in the hyperintensity area surrounding the lesion. After steroid administration, the T2-weighted image at 11 months shows a decrease in the hyperintensity area. The diffusion-weighted image and ADC map at 11 months reveal a hyperintensity area and notably low ADC in the right lateral region of the midbrain. Radiation necrosis usually presents as mixed signal intensity patterns on diffusion-weighted images, often with marked hypointensity, and these regions correspond to high ADC values [1]. However, radiation necrosis with notably low ADC values has been reported due to the high viscosity and cellular composition of the inflammatory infiltrate in early necrosis and scarring from gliosis or fibrosis in later phases [2].


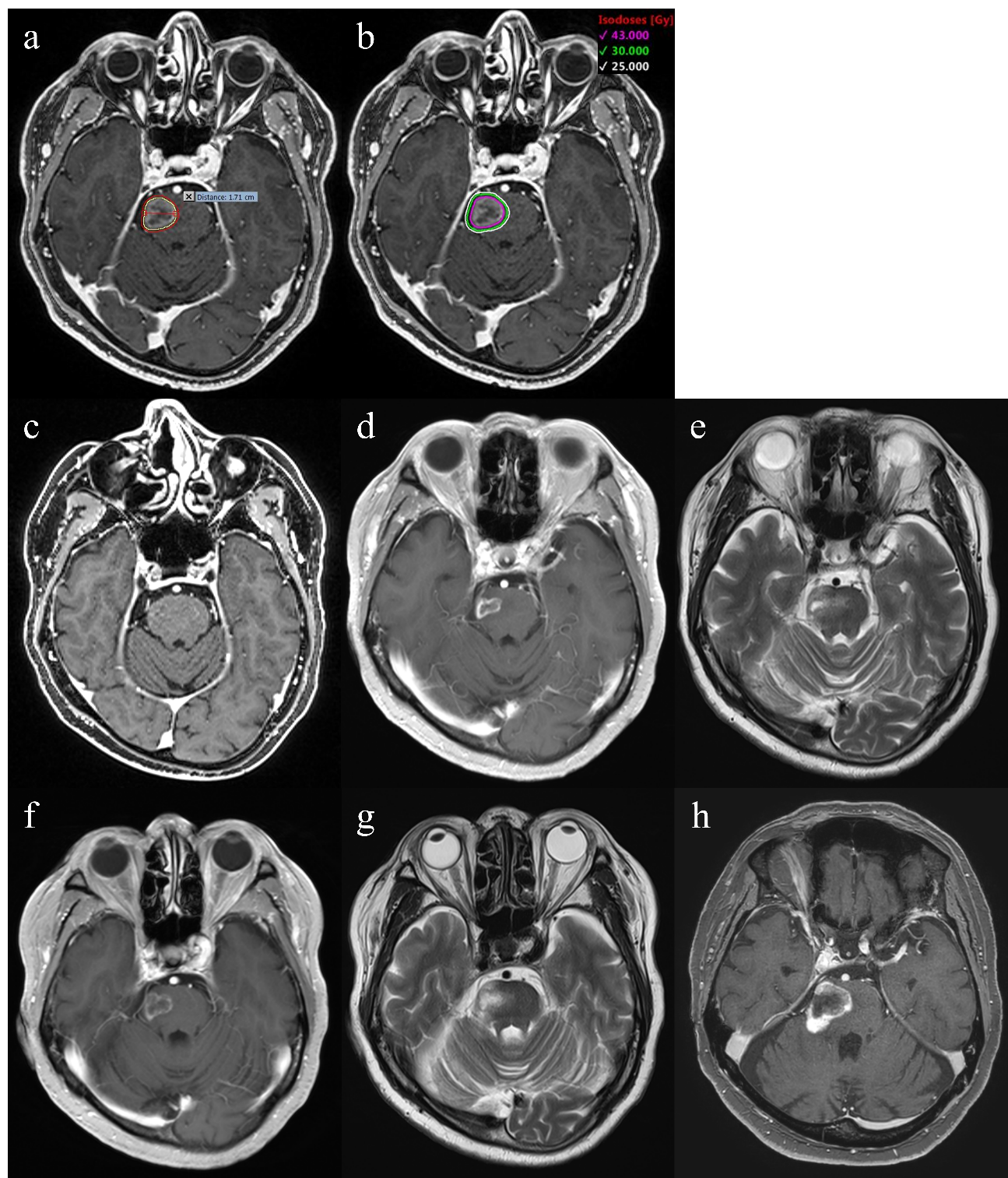


**Supplementary Fig. 4.** A case of pontine metastasis from small cell lung cancer treated with stereotactic radiotherapy (SRT) of 30 Gy in five fractions, presenting with suspected local failure.

(a) Pre-SRT axial contrast-enhanced (CE)-T1-weighted image showing the GTV (yellow line) and PTV (red line).

(b) Dose distribution of SRT, depicting 43 Gy (purple line), 30 Gy (green line), and 25 Gy (white line).

(c) CE-T1-weighted image acquired 4 months post-treatment.

(d), (e) CE-T1 and T2-weighted images acquired 6 months post-treatment (under steroid treatment).

(f), (g) acquired 8 months post-treatment (under an increased steroid dosage).

(h) CE-T1-weighted image acquired 11 months post-treatment.

The CE-T1-weighted image at 4 months post-treatment shows a decrease in lesion size with reduced contrast enhancement compared with the pre-SRT image; however, the CE-T1-weighted image at 6 months shows lesion growth with an enhancing rim, and the T2-weighted image demonstrates a corresponding distinct margin, indicating a T1/T2 match [3], except for the inner edge. The T2-weighted image shows hyperintensity areas around the lesion. Despite an increased steroid dosage, the lesion further increases in size on CE-T1 and T2-weighted images at 8 months. The CE-T1-weighted image at 11 months shows further enlargement. Both recurrence and adverse radiation effects are considered mixed, but based on imaging findings and clinical course, recurrence is considered predominant.

References

1. Asao C, Korogi Y, Kitajima M et al. Diffusion-weighted imaging of radiation-induced brain injury for differentiation from tumor recurrence. *AJNR Am J Neuroradiol* 2005;26:1455–60.

2. Katsura M, Sato J, Akahane M et al. Recognizing radiation-induced changes in the central nervous system: Where to look and what to look for. *Radiographics* 2021;41:224–48.

3. Kano H, Kondziolka D, Lobato-Polo J et al. T1/T2 matching to differentiate tumor growth from radiation effects after stereotactic radiosurgery. *Neurosurgery* 2010;66:486–91; discussion 491-2.
